# *Trichomonas vaginalis* Infection and Associated Risk Factors in a Socially-Marginalized Female Population in Coastal Peru

**DOI:** 10.1155/2009/752437

**Published:** 2009-06-29

**Authors:** Segundo R. Leon, Kelika A. Konda, Kyle T. Bernstein, Jose B. Pajuelo, Ana M. Rosasco, Carlos F. Caceres, Thomas J. Coates, Jeffrey D. Klausner

**Affiliations:** ^1^Laboratorio de Salud Sexual, Laboratorios de Investigación y Desarrollo, Universidad Peruana Cayetano Heredia, Lima 31, Peru; ^2^Unidad de Salud, Sexualidad y Desarrollo Humano, Facultad de Salud Pública y Administración, Universidad Peruana Cayetano Heredia, Lima 31, Peru; ^3^Division of Infectious Diseases and Program in Global Health, David Geefen School of Medicine, University of California, Los Angeles, CA 90024, USA; ^4^San Francisco Department of Public Health, STD Prevention and Control Section, San Francisco, CA 94103, USA

## Abstract

*Objective.* The epidemiology of *Trichomonas vaginalis* infection among sexually active socially-marginalized women in three urban, coastal Peruvian cities was examined in order to quantify the prevalence of trichomonas infection and identify associated risk factors. *Methods*. We conducted a cross-sectional, venue-based study of women from socially-marginalized populations in three coastal Peruvian cities. *Results*. Among the 319 women enrolled, the overall prevalence of trichomonal infection was 9.1% (95% CI, 5.9%–12.3%). The mean age was 26.3 years, and 35.5% reported having had unprotected intercourse with nonprimary partners and 19.8% reported two or more sex partners in the last three months. Trichomonal infection was associated with increased number of sex partners (PR 2.5, 95% CI 1.4–4.6) and unprotected sex with nonprimary partner in the last three months (PR 2.3, 95% CI 1.1–4.9). *Conclusions*. A moderately high prevalence of trichomonal infection was found among women in our study. Trichomonal infection was associated with unprotected sex and multiple sex partners. Efforts to control the continued spread of trichomonal infection are warranted.

## 1. Intoduction

*Trichomonas vaginalis * infection is one of the most common curable sexually transmitted infections [1]. WHO estimates that 173 million of new cases occur annually [2]. Trichomonal infection has been associated with adverse reproductive health outcomes including pelvic inflammatory disease, low infant birth weight and premature delivery [3–6]. Untreated infection can persist up to 5 years [7]. Furthermore, Trichomoniasis has been shown to increase transmission and acquisition of other sexually transmitted diseases, including HIV infection [1, 8, 9]. The frequency of trichomonal infection is not monitored in most countries nor do control programs exist. Peruvian STI guidelines do not include trichomonas diagnostic testing; the syndromic management of suspected vaginal trichomoniasis is the current standard for clinical care [10]. Trichomonal infection is most likely more common among women and men than is generally understood, and long standing, asymptomatic infections may lead to continued community level transmission. As Trichomonas prevalence depends on many factors such as age, sexual activity, and the number of sex partners, a better understanding of the epidemiology of this infection in women is needed to foster disease control programs in populations at risk for reproductive health complications.

## 2. Methods

### 2.1. Study Design and Participant Enrollment

This study was a cross-sectional analysis of the behavioral and biological data from the baseline assessment of a cohort subsequently enrolled in the NIMH Collaborative HIV/STD Prevention Trial collected from 2003 to 2005 in Peru [11]. We enrolled participants from low-income neighborhoods in three coastal Peruvian cities: Lima, Trujillo, and Chiclayo. We chose neighborhoods based on data from the Peruvian National Institute of Statistics and Informatics [12]. Study staff visited selected neighborhoods to recruit socially marginalized women. Socially marginalized women were characterized through informal interviews and participant observation, and based on ethnographic information collected prior to the study baseliness were shown to have high levels of sexual risk behaviors. We defined *socially marginalized women * as those women living in low-income neighborhoods “who contradicted Peruvian social norms by spending time with and having sex with poor unemployed men.” Most of these women were also unemployed, and although the majority reported a stable sex partner, most of them also reported having multiple sex partners, further defying the social norms of Peru [13]. 

Eligible women were recruited by outreach workers from venues such as volleyball fields, hair salons, parks, bars, and street corners. The number of venues selected per city was approximately proportional to the city's overall population. Eligible participants were restricted to women between 18 and 40 years of age who reported having had sex in the past six months, intended to stay in the neighborhood for at least two years, and who frequented the target venue at least twice a week.

All eligible women who signed informed consent were enrolled.

### 2.2. Data Collection

Participants were invited to a temporary project office in their neighborhood, where we privately administered a 30-minute structured survey in Spanish, wherein trained interviewers read questions to participants and entered their answers into a portable computer. The survey instrument collected sociodemographic information as well as detailed sexual risk behavior with the last five sex partners, number of sex partners, STI-related symptoms, alcohol and drug, use and HIV status.

Upon completing the survey, participants underwent pretest counseling for STIs including HIV infection, conducted by a trained counselor. Trained laboratory technicians then explained to each participant how to self-collect a vaginal specimen using a dacron swab. A second dacron swab was stored for *Chlamydia trachomatis * and *Neisseria gonorrhoeae* PCR testing (CT/NG Amplicor PCR, Roche Diagnostics, NJ, USA), and a blood sample was also collected for HIV-1 (Genetic Systems Bio-Rad Laboratories, Hercules, Calif, USA), Herpes Virus Simplex Type 2 antibodies (HerpeSelect; Focus Technologies, Cypress, Calif, USA), and syphilis testing (RPRnosticon, Shield Diagnostics, Dundee, UK and Serodia-TPPA, Fujirebio Diagnostics Inc, Tokyo, Japan). Participants returning for results underwent posttest counseling to ensure their understanding of the meaning of both positive and negative results and for treatment in case of a positive result. Treatment for trichomoniasis consisted of 2 g, single dose metronidazole by mouth administered by trained health personnel. All participants with positive results were encouraged to share test results with recent sex partners. Reimbursement for travel costs and time spent during the assessment was given to each participant at the rate of 15 Peruvian Soles (approximately $5) at their first visit and an additional 10 Peruvian Soles (approximately $3) at their posttest visit.

### 2.3. Laboratory Methods for Trichomonas vaginalis Detection

All vaginal swab specimens were immediately inoculated and transported at ambient temperature using the In Pouch TV culture media (Biomed Diagnostics, White City, Ore, USA) to the Cayetano Heredia University—Sexual Health Laboratory in Lima, Chiclayo, or Trujillo. Cultures were transported to the laboratory within 6–8 hours after sample collection and incubated at 37°C once they arrived at the laboratory. To monitor the performance of test and as part of the quality control of the study, known positive samples were inoculated in the field, transported, and cultured under the same conditions. All samples, including the controls, were observed microscopically at 400x power for the presence of motile, oval flagellated protozoan daily for 5 days. The observation of motile trichomonads was reported as positive, and specimens showing no organisms after 5 days of incubation were considered negative for *T. vaginalis.*

Laboratory work was performed in accordance with the College of American Pathology Quality Assurance and Quality Control program.

### 2.4. Data Analysis

Trichomonal infection was the primary outcome of interest. Explanatory variables included demographic characteristics and sexual and drug using behaviors. Categorization of continuous variables was based on the distribution of data. As odds ratios overestimate the estimated risk when the prevalence is high, we calculated prevalence ratios to avoid that bias [14]. To model the prevalence ratios we used Poisson family generalized linear models and adjusted the standard errors to account for the clustered nature of sample. Multivariate models were created taking into account all variables that were significant at *P*-value <.1 in bivariate analysis [14]. The variables added to the multivariate model were selected based on likelihood ratio tests comparing nested to saturated models and included only if the likelihood ratio test had a *P*-value <.05. All statistical analyses were conducted in Stata 9.2 (College St. Tex, USA).

### 2.5. Human Subjects

The study was approved by the Committee on Human Research of the University of California, San Francisco; University of California, Los Angeles; Naval Medical Research Center Detachment and Cayetano Heredia University of Peru. We collected data only from participants that gave written informed consent to participate in the study.

## 3. Results

Between 2003 and 2005, we recruited and assessed 308 women from 20 low-income communities in coastal Peru. The overall prevalence of *T *. *vaginalis * infection by culture was 9.1% (95% CI, 6.1%–12.9%). The median age of the women was 24 years (IQR, 21–31). Demographic and risk behavior characteristics are described in detail in Table 1. 

**Figure 1:**
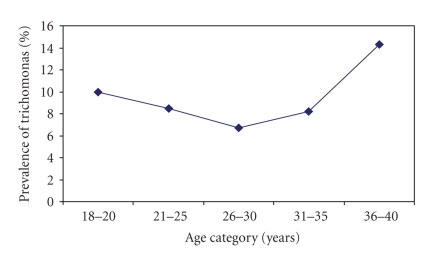
Prevalence of trichomonas among socially-marginalized women by age category in urban coast Peru, 2003–2005.

Women with *T. vaginalis * infection were more likely to be older; the highest prevalence was found in the oldest age group, 36–40 years (Figure 1). Those with less education were 3 times more likely to have trichomonal infection compared to those who completed high school, and being single was associated with having trichomonal infection (Table 1). The prevalence of trichomonal infection among women with vaginal discharge and painful urination in the prior six months was lower than the prevalence among those who did not report vaginal discharge during the same period, although the difference was not statistically significant. Women with HSV-2 infection and syphilis infection were twice as likely to have trichomonal infection. In the multivariate model, because only the number of partners in the past 6 months significantly improved the model, no other variables were entered into the model.

## 4. Discussion

We found a moderately high prevalence of *T *. *vaginalis * infection among socially-marginalized young women from low-income communities in coastal Peru. *T. vaginalis * infection occurred more often among older women (See Figure 1), women who had not completed high school, single women, women who reported unprotected sex with a nonsteady partner, and women who reported multiple sex partners. No association was found between any vaginal symptoms and *T. vaginalis*.

While most epidemiological studies on trichomonal infection have been focused on pregnant women, adolescents, and female sex workers accessing care, there are few studies at the population-level and in particular, socially-marginalized women [5, 15–19]. Our findings are important because such hard to reach populations are often under-represented because they are not seeking health care and represent a high-risk target population and potential core population that maintain endemic trichomonal infections at the population level. In Latin America trichomonal prevalence has varied from 2.9% to 16.5%, where the highest prevalence was found in population samples in the jungle and the highlands of Perú, and the lower prevalences were found in clinical settings such as family planning clinics, pharmacies, and obstetrics services. In general, tested populations in those studies were most often symptomatic women [15, 20, 21].

We found a strong association between trichomonal infection and increased age. The association between higher prevalence of trichomonal infection and older age is unusual for nonviral sexually transmitted infections. Chlamydia and gonorrhea are more prevalent in younger female populations. The increased prevalence of trichomonal infection in older women suggests long-term prevalent infection that does not spontaneously resolve and would be missed by screening programs focused on younger age women [22].

The prevalence of trichomonal infection was not different between women with or without presence of recent painful urination or vaginal discharge. That finding suggests that syndromic management of *T. vaginalis * may result in both over and under treatment. The asymptomatic nature of trichomonal infection also could contribute to a long duration of infection and continued spread of infection in the population [1, 7, 23]. Our data, and others [4, 21, 24], attest to the asymptomatic nature of *T. vaginalis * and the need for screening and treatment programs to control the spread of trichomonal infection. Results from other Peruvian studies as well as the results in the present study indicate that there is no direct relationship between *T. vaginalis * infection and symptoms making syndromic treatment unlikely to impact the population burden of infection [20, 21]. 

Our analysis had several limitations. First, the sample size was small resulting in limited power to assess multivariate models between demographic and behavioral characteristics and *T. vaginalis*. Furthermore, the results presented here may not be generalizable to other female populations. While response bias is always a concern, we believe that the use of standardized interviews by trained staff helped to minimize social desirability and other information biases. Importantly as all epidemiologic information was collected prior to testing, there was no reason to suspect differential bias associated by trichomonal infection. Selection bias was also a concern, but because most women were asymptomatic we do not believe that those with potential trichomonal infection were more likely to participate compared with those without infection.

Since *T. vaginalis * is a common sexually transmitted pathogen, the screening and treatment of sex partners of infected women must be also prioritized as a public health measure to prevent reinfection and reduce infection prevalence and complications in untreated males [23, 25]. As the majority of the assessed population in this study was in a fertile age range, our findings support the need for improved disease control activities to reduce adverse trichomoniasis-associated reproductive health outcomes such as ectopic pregnancy, low infant birth weight, preterm labor, and non-HPV associated cervical neoplasia [14, 26, 27]. As *T. vaginalis* is widely prevalent and one of the easiest infections to treat with an inexpensive antimicrobial, further studies are needed to determine if screening and treatment of trichomoniasis will improve the reproductive health and birth outcomes of women.

## Figures and Tables

**Table 1:** Demographics, risk factors, and prevalence of *T. vaginalis* infection among women from low-income communities of urban coastal Peru, 2003–2005 (*N* = 319).

	No. (%)	*Trichomonas vaginalis* prevalence (%)	Prevalence ratio	*P*-value
Demographics				

Reported age (years)				
18–20	75 (23.5)	10.7	1	
21–25	100 (31.4)	8.0	0.8 (0.3–2.3)	0.61
26–30	59 (18.4)	6.8	0.6 (0.2–2.2)	0.49
31–35	50 (15.7)	8.0	0.8 (0.2–2.7)	0.67
36–40	35 (11.0)	14.3	1.3 (0.5–3.9)	0.59
High school education				
Yes	129 (40.6)	7.8	1	
No	189 (59.4)	10.1	1.3 (0.9–1.9)	0.171
Marital status				
Married	162 (50.8)	6.8	1	
Single	115 (36.0)	13.9	2.0 (0.9–4.5)	0.08
Other (separated, widowed, divorced)	42 (13.2)	4.8	0.7 (0.2–3.2)	0.65

Risk behavior				

Years sexual activity				
0–5	94 (30.5)	9.7	1	
6–10	89 (28.9)	6.7	0.7 (0.3–1.8)	0.45
11–15	58 (18.8)	8.6	0.9 (0.4–2.1)	0.79
16–20	39 (12.7)	10.3	1.1 (0.3–3.5)	0.92
21-more	28 (9.1)	14.3	1.5 (0.6–3.6)	0.38
Number of sex partners in last six months				
0-1	220 (71.4)	6.4	1	
2+	80 (28.6)	15.9	2.5 (1.4–4.6)	<0.01
Months living with partner				
0–6 months	17 (11.26)	17.65	3.4 (0.9–13.3)	0.08
6–12 months	134 (88.74)	5.97	1	
Smoked cocaine paste, 30 days				
No	309 (96.9)	8.4	1	
Yes	10 (3.1)	30.0	3.6 (1.3–10.1)	0.02
Any sex last 3 months with nonsteady partner				
No	193 (60.7)	6.2	1	
Yes	125 (39.3)	12.8	2.1 (1.0–4.3)	0.06
Unprotected sex last 3 months with nonsteady partner				
No	209 (65.7)	6.7	1	
Yes	109 (34.3)	13.8	2.1 (1.1–4.0)	0.04

Symptoms				

Episode of painful urination in the last 6 months				
No	185 (58.0)	9.7	1	
Yes	134 (42.0)	8.2	0.8 (0.4–1.7)	0.63
Episode of vaginal discharge in the last 6 months				
No	215 (67.6)	10.7	1	
Yes	103 (32.4)	5.8	0.5 (0.2–1.8)	0.32

STIs				

HIV-1				
No	318 (99.7)	9.1		
Yes	1 (0.3)	0.0	Undefined	
HSV-2				
No	179 (57.9)	6.7	1	
Yes	130 (42.1)	13.1	2.0 (1.1–3.5)	0.03
Syphilis				
No	307 (96.2)	8.8	1	
Yes	12 (3.8)	16.7	1.9 (0.8–4.5)	0.15
Chlamydia				
No	274 (85.9)	8.4	1	
Yes	45 (14.1)	13.3	1.6 (0.8–3.0)	0.15
Gonorrhea				
No	306 (97.1)	9.2		
Yes	9 (2.9)	0.0	Undefined	
